# Test-treat-track-test-treat (5T) approach for *Schistosoma haematobium* elimination on Pemba Island, Tanzania

**DOI:** 10.1186/s12879-024-09549-w

**Published:** 2024-07-02

**Authors:** Lydia Trippler, Lyndsay Taylor, Mohammed Nassor Ali, Sarah Omar Najim, Khamis Seif Khamis, Jan Hattendorf, Saleh Juma, Shaali Makame Ame, Fatma Kabole, Said Mohammed Ali, Stefanie Knopp

**Affiliations:** 1 Swiss Tropical and Public Health Institute, Allschwil, Switzerland; 2 University of Basel, Basel, Switzerland; 3 Ivo de Carneri, Wawi, Chake Chake, Pemba, United Republic of Tanzania; 4 Neglected Diseases Program, Zanzibar Ministry of Health, Mkoroshoni, Pemba, United Republic of Tanzania; 5 Neglected Diseases Program, Zanzibar Ministry of Health, Lumumba, Unguja, United Republic of Tanzania

**Keywords:** Interventions, Case finding, Elimination, Interruption of transmission, Mass drug administration, Schistosomiasis, *S. haematobium*, Surveillance-response, Test-and-treat, Test-treat-track-test-treat, Zanzibar

## Abstract

**Background:**

After decades of praziquantel mass drug administration (MDA), several countries approach schistosomiasis elimination. Continuing MDA in largely uninfected populations no longer seems justified. Alternative interventions to maintain the gains or accelerate interruption of transmission are needed. We report results, strengths, and shortcomings of novel test-treat-track-test-treat (5T) interventions in low *Schistosoma haematobium* prevalence areas on Pemba, Tanzania.

**Methods:**

School- and household-based surveys were conducted in 2021 and 2022 to monitor the *S. haematobium* and microhematuria prevalence and assess the impact of interventions. In 2021, 5T interventions were implemented in 15 low-prevalence areas and included: (i) testing schoolchildren in primary and Islamic schools for microhematuria as a proxy for *S. haematobium*, (ii) treating positive children, (iii) tracking them to their households and to water bodies they frequented, (iv) testing individuals at households and water bodies, and (v) treating positive individuals. Additionally, test-and-treat interventions were implemented in the 22 health facilities of the study area.

**Results:**

The *S. haematobium* prevalence in the school-based survey in 15 low-prevalence implementation units was 0.5% (7/1560) in 2021 and 0.4% (6/1645) in 2022. In the household-based survey, 0.5% (14/2975) and 0.7% (19/2920) of participants were infected with *S. haematobium* in 2021 and 2022, respectively. The microhematuria prevalence, excluding trace results, in the school-based survey was 1.4% (21/1560) in 2021 and 1.5% (24/1645) in 2022. In the household-based survey, it was 3.3% (98/2975) in 2021 and 5.4% (159/2920) in 2022. During the 5T interventions, the microhaematuria prevalence was 3.8% (140/3700) and 5.8% (34/594) in children in primary and Islamic schools, respectively, 17.1% (44/258) in household members, and 16.7% (10/60) in people at water bodies. In health facilities, 19.8% (70/354) of patients tested microhematuria-positive.

**Conclusions:**

The targeted 5T interventions maintained the very low *S. haematobium* prevalence and proved straightforward and feasible to identify and treat many of the few *S. haematobium*-infected individuals. Future research will show whether 5T interventions can maintain gains in the longer-term and expedite elimination.

**Trial registration:**

ISRCTN, ISCRCTN91431493. Registered 11 February 2020, https://www.isrctn.com/ISRCTN91431493.

**Supplementary Information:**

The online version contains supplementary material available at 10.1186/s12879-024-09549-w.

## Background

Human schistosomiasis is a neglected tropical disease (NTD) that can cause serious health consequences. Currently, people from 78 countries are at risk of an infection with *Schistosoma* spp., primarily in sub-Saharan Africa [1, 2]. The primary intervention against schistosomiasis in endemic countries is preventive chemotherapy through mass drug administration (MDA) of praziquantel, implemented by national schistosomiasis control programs [1, 3]. Regular MDA is likely the main driver for the significant reduction in prevalence in sub-Saharan Africa over the past 20 years [4].

Several countries, including Egypt and Morocco [5–8], and certain regions in sub-Saharan Africa, for example in Cameroon [9] and Côte d’Ivoire [10] have now reached very low levels of schistosomiasis and are approaching elimination. Large-scale MDA no longer seems justified in these areas as the majority of the population is not infected with *Schistosoma* spp [11, 12]. Providing praziquantel to uninfected people would be a misuse of resources that are urgently needed elsewhere, specifically in regions where children and adults still suffer from high morbidity. Yet, there is a lack of clear guidelines and evidence at what prevalence levels it is safe to stop praziquantel MDA and what interventions can be used as alternatives. Such alternative interventions should ideally not only sustain the gains made by multiple rounds of MDA and prevent resurgence, but support countries to progress towards the ultimate goal: interruption of transmission.

To reach interruption of transmission, current WHO guidelines recommend that in communities where the prevalence of schistosomiasis is < 10%, programs either continue with MDA at the same or reduced frequency or use a clinical approach of test-and-treat if there had not been a program of regular preventive chemotherapy before [3]. However, these recommendations are conditional since the certainty of evidence is still very low [3]. More studies need to be conducted to show if MDA at reduced frequency or test-and-treat approaches indeed can support the elimination goals.

On the Zanzibar islands, Unguja and Pemba, urogenital schistosomiasis caused by infection with *Schistosoma haematobium* used to be a considerable public health problem [11]. However, small and large-scale interventions against schistosomiasis have been implemented for almost 100 years and contributed to a substantial decrease in the prevalence and morbidity [11]. Since 2012, the overall *S. haematobium* prevalence in Zanzibar is below 10% [13–15]. To reach elimination, annual or biannual MDA with praziquantel for children and adults is implemented in schools and communities across the islands since 2013 [13–15]. Additionally, snail control and behavior change communication, are applied in large-scale operational research projects, such as the Zanzibar Elimination of Schistosomiasis project (2012–2017) and the SchistoBreak study (2020–2024) [16, 17]. Since 2017, the islands have reached ‘elimination as a public health problem’ and aim to proceed towards ‘interruption of *Schistosoma* transmission’ [11, 13, 18]. In 2020, the overall *S. haematobium* prevalence in Zanzibar was 3.4% in schoolchildren and 0.4% in adults [15]. However, despite the very low overall prevalence, there is a marked heterogeneity of transmission and infection across each island, with many low-prevalence and a few moderate to high prevalence administrative areas [15]. The Zanzibar islands are now facing the challenges of how to address this heterogeneity, of whether, when, and where to stop MDA and of what interventions to use as alternatives to large-scale MDA to maintain the gains or to reach interruption of transmission.

The SchistoBreak study, implemented from 2020 to 2024 in the north of Pemba, aims to address this spatial heterogeneity by targeting different sets of elimination interventions to the local micro-epidemiology of *S. haematobium* [17]. In low-prevalence areas, a surveillance-response approach is implemented as an alternative intervention to MDA. Part of this surveillance-response approach are targeted test-treat-track-test-treat (5T) interventions, where students are tested for urogenital schistosomiasis, treated if positive, and tracked to their homes and water bodies, where household members and people at water bodies, respectively, are also offered testing and treatment if positive. Here, we aimed to assess whether 5T interventions can maintain or further reduce the *S. haematobium* prevalence in low-prevalence areas and report strengths and shortcomings from the first period of targeted 5T interventions implemented in 15 low-prevalence areas in 2021.

## Methods

### Study setting

The SchistoBreak study is conducted on Pemba, one of the two main islands of the Zanzibar archipelago, United Republic of Tanzania. Pemba is located 30 km off the east coast of the country’s mainland. The island is divided into four districts, “Mkoani”, “Chake Chake”, “Wete”, and “Micheweni”, which are subdivided into 129 small administrative areas known as “shehias” [19]. The SchistoBreak study area consists of 20 shehias, which are referred to in this publication as “implementation units” (IUs). The 20 IUs are located in the two northern districts of Pemba, Wete and Micheweni, which are characterized by their mostly rural environment and the presence of numerous water bodies [18]. Recent data from a national census conducted in 2022 indicate a total population of 543,441 for Pemba, with 272,091 people living in the districts Wete and Micheweni [20]. The vast majority of Pemba’s population is of the Islamic faith, and it is common for children to attend madrassas (Islamic schools) in the afternoons or on weekends, in addition to attending primary or secondary school. There are 47 health facilities located in the districts Wete and Micheweni, including primary health care units (PHCUs) and hospitals. Of these, 22 are located within the SchistoBreak study area [20]. On Pemba, only urogenital schistosomiasis caused by *Schistosoma haematobium* is endemic, while autochthonously acquired intestinal schistosomiasis is absent [11].

### Study design and participants

The SchistoBreak study follows a longitudinal design with three annual intervention periods and four annual school-based and household-based surveys that employ a cross-sectional sampling approach to monitor the *S. haematobium* prevalence in the IUs and to measure the impact of the interventions [17]. The study runs over four years, from February 2020 to March 2024. Annual surveys are conducted from November to February/March each year, before and after the intervention periods from May to October [17]. Different sets of interventions targeted to the local micro-epidemiology are employed. In IUs with a *S. haematobium* prevalence of ≥ 3% in schoolchildren and ≥ 2% in communities, a combination of annual MDA, snail control, and behavior change interventions is implemented to accelerate the interruption of *S. haematobium* transmission in “hotspots”. In IUs with a *S. haematobium* prevalence of < 3% in schoolchildren and < 2% in communities, no MDA is provided. Instead, surveillance-response interventions, including the 5T interventions described here, are implemented in schools, households, and at water bodies as described below. Furthermore, in health facilities within all IUs in the study area a simple test-and-treat approach is conducted.

Any person aged ≥ 4 years old enrolled in one of the study schools/madrassas or living in the study area is eligible to participate in the annual surveys and the interventions. Children < 4 years old are excluded from the study as it is challenging to collect urine samples from babies and toddlers and as the safety of praziquantel (BILTRICIDE^®^) has been established only for individuals aged ≥ 4 years [21].

### School-based and household-based surveys

From November 2020 to February 2021 and from November 2021 to March 2022, school-based and household-based surveys were implemented in the 20 IUs of the study site. The school-based surveys were conducted in each largest public primary school within the 20 IUs, and the household-based surveys were conducted in each community within the 20 IUs. No school-based survey was conducted if an IU did not have a public primary school.

In each study school, 175 children from nursery school through grade six were randomly selected to participate. The random sampling is described in detail elsewhere [18]. Briefly, a total of 25 children were randomly selected from each grade (nursery school to grade six), balanced by sex. Subsequently, the selected children were registered with demographic details and given consent and assent forms. On the following day, upon return of the signed consent and assent forms, the present children were given a transparent plastic container (100 ml) labeled with a unique identifier code and asked to provide a fresh urine sample.

In each study community, 70 housing structures (in 2021) or 80 housing structures (in 2022), respectively, were randomly preselected. The process used to randomize and locate the housing structures within the communities is described elsewhere in detail [18, 22]. Briefly, a navigation system installed on mobile devices was used to locate each housing structure in the community. If a housing structure was inhabited, after consenting, an adult person living in the house was invited to participate in a questionnaire interview and to disclose sociodemographic details of all household members. Furthermore, all eligible household members were invited to participate in the study by signing an informed consent form and providing a urine sample for examination. Urine samples were provided either immediately at the first visit, or in case of absent household members, until the second visit on the following morning or, if still absent, until the third visit on the third day.

### Test-treat-track-test-treat interventions

The first period of 5T interventions was implemented from June 2021 to October 2021 in 15 IUs that had met the criteria of a *S. haematobium* prevalence of ≤ 3% in the school-based survey and of ≤ 2% in the household-based survey in the baseline survey in 2021 [17, 18]. The 5T interventions consist of five (5) parts (testing, treating, tracking, testing, and treating). The first and second parts (test, treat) were implemented in schools and madrassas. Since children enrolled in grades 3–5 in Zanzibar are at the highest risk of infection [15], the initial testing to identify as many positives as possible involved all children in grades 3–5 in one primary school and all children in one madrassa, respectively, per low-prevalence IU. No primary school was located in four of the 15 IUs. Here, only children from madrassas were tested. Before testing, all children were registered with demographic details. In madrassas, children were additionally asked which primary/secondary school they attended, in which grade they were enrolled, and whether they had already been tested (and treated) within the same 5T intervention period to avoid double testing and treating. The following day, once children had submitted the signed consent and assent forms and produced their urine sample between 9 am and 4 pm (depending on school times), the samples were tested for microhematuria as a proxy for *S. haematobium* infection at the point-of-care directly in the schools by the trained field enumerators, using Hemastix reagent strips (Hemastix; Siemens Healthcare Diagnostics AG; Zurich, Switzerland). In the second part (treat), all children who tested microhematuria-positive were treated with a single dose of praziquantel (40 mg/kg body weight) by a member of the Zanzibar Neglected Diseases Program, who was part of our research team, using a dose-pole [23]. Next, in the third part (track), all positive-tested children were accompanied to their homes and to water bodies they frequently used. Water bodies located outside the study area were not visited. Our assumption was that household members of positive children may use the same water bodies where transmission occurs and, as other people that use these water bodies, be at high risk of infection. In the fourth and fifth parts (test, treat), all individuals present at the time of the visit at the homes and the water bodies were invited to respond to demographic questions and to provide a fresh urine sample after written consent. Both households and water bodies were visited once. The study team spend up to two hours at each water body to invite present or arriving individuals to test and treat activities. Each urine sample was tested for microhematuria on site by the trained field enumerators and subsequently taken to the Public Health Laboratory – Ivo de Carneri (PHL-IdC) in Chake Chake, Pemba, for *S. haematobium* egg detection (see below). If a household member or individual present at a water body tested positive for microhematuria and/or *S. haematobium* eggs, the individual was offered immediate treatment with praziquantel (40 mg/kg body weight) on site (if positive for microhematuria) or called by phone and encouraged to seek treatment at the nearest health facility (if positive for *S. haematobium* eggs by urine filtration).

### Test-and-treat in health facilities

In June 2021, staff from each of the 22 health facilities (20 PHCUs and two hospitals) in the study area were invited to the PHL-IdC for a meeting, including a presentation about the life cycle, symptoms, and health consequences of urogenital schistosomiasis, discussion, and training on the use of Hemastix reagent strips to test for microhematuria in urine samples. Subsequently, the staff were equipped with Hemastix reagent strips to test for microhematuria in patients presenting with symptoms consistent with urogenital schistosomiasis, such as visible hematuria, painful and/or frequent urination, abdominal pain, or infertility within the routine services of their health facility. They were also equipped with praziquantel to treat individuals who tested microhematuria-positive. In addition, the staff were requested to keep a record of patients presenting with schistosomiasis-related symptoms, were tested for microhematuria and treated with praziquantel (40 mg/kg body weight) if positive. These records were collected every two weeks from July 2021 onwards by a member of the SchistoBreak study team.

### Laboratory examinations

All urine samples collected during the surveys and at the households and water bodies during the 5T intervention period were transported to the PHL-IdC. Here, 10 ml of each urine sample were filtered through a 13 mm fabric filter (Sefar Ltd., Bury, UK), which was placed in a Swinnex plastic filter holder (Millipore-Merck KGaA, Darmstadt, Germany) attached to a plastic syringe. The filters were placed on a microscope slide labeled with the participant’s unique identifier code and examined under a light microscope for the presence and number of *S. haematobium* eggs. In addition, all urine samples collected during the surveys were examined for microhematuria on the same day using Hemastix reagent strips. All examinations in the laboratory were conducted by three experienced laboratory technicians who recorded the results for each identifier code on case report forms. As the urine samples collected during the 5T interventions were already tested for microhematuria at the point-of-care in the field by trained field enumerators, the testing was not repeated in the laboratory.

### Data management

All demographic data collected in the school-based survey and all data collected during the questionnaire interviews in the household-based survey were captured by inserting responses into the preprogrammed questionnaire application Open Data Kit (ODK, www.opendatakit.org), installed on Samsung Galaxy Tab A tablets. All data containing results of Hemastix reagent strip examinations conducted at the point-of-care in schools, households, or at water bodies during the 5T intervention period were also captured through ODK. These electronic data were subsequently sent to the ODK server, hosted by the Swiss Tropical and Public Health Institute (Swiss TPH) in Allschwil, Switzerland. All results of laboratory examinations of urine samples, including the results of urine filtrations and Hemastix reagent strips collected during the surveys, were recorded on paper and then double-entered into a Microsoft Excel spreadsheet (2016, version 16.0.5395.1000) by two experienced data entry clerks from PHL-IdC. These spreadsheets were sent to the Swiss TPH via a secure server and cleaned using R version 4.0.3 (www.r-project.org) and Stata/IC 16 (StataCorp LLC, College Station, TX, USA). Mismatching double-entry results were checked against the original paper forms and reentered correctly. For statistical analysis, the laboratory results were merged with the questionnaire and registration data based on the unique identifier codes. To inform participants about their *S. haematobium* infection status, participant names were merged once with laboratory analysis data. Otherwise, names were kept in a separate file, and only coded data were used for statistical analyses.

### Statistical methods

Statistical analyses for this publication were conducted using R version 4.1.3.

Only data collected in IUs classified as low-prevalence IUs during the baseline survey of the SchistoBreak study [18] were included in the analysis of this publication. One exception are the data collected in the 22 health facilities that were spread across the study area. All data from patients in health facilities were included in the analysis when patients were tested for microhematuria between July 2021 (the start of the collaboration with health facilities) and November 2021 (the end of the first intervention period).

All eligible individuals who provided a urine sample were included in the parasitological analyses of the surveys, the 5T interventions, and the test-and-treat interventions in health facilities. A participant was considered microhematuria-positive if the result of Hemastix reagent strips was trace, small (+), moderate (++), or large (+++) according to the color chart provided by the manufacturer. A participant was considered positive for *S. haematobium* if one or more eggs were detected by urine filtration.

Prevalence of microhematuria and *S. haematobium* in the surveys, 5T interventions, and test-and-treat interventions in health facilities was calculated overall and per IU for each survey or intervention period, respectively. The statistical significant difference of prevalence between the survey years was calculated for each survey using a two-sample proportion test.

For children tested in madrassas, the children’s potential additional enrolment in a primary or secondary school and their grade level were assessed to determine how many children were captured in madrassas but were not part of the 5T interventions in primary schools. Moreover, the microhaematuria prevalence of madrassa students that also attended a public school *versus* non-school attendees was determined.

The sites of data collection during the 5T interventions, including screened schools and madrassas and tracked households and water bodies, were mapped with ArcMap version 10.6.1 (ESRI, Redlands, CA, USA). The locations of households were geographically masked in order to preserve confidentiality.

## Results

### Participation in school-based and household-based surveys 2021 and 2022

The number of children and household members enrolled and the overall results of the baseline school-based and household-based survey, respectively, in all 20 IUs carried out from November 2020 to February 2021 are presented elsewhere [18]. This baseline survey revealed a total of five hotspot IUs according to the pre-set prevalence threshold criteria. Additional 15 IUs were identified as low-prevalence, including 11 low-prevalence primary schools (no primary school was located in four IUs) and 15 low-prevalence communities. Subsequent results focus on these 11 low-prevalence primary schools and 15 low-prevalence communities, since the 5T interventions that are the primary focus of this publication were only implemented in the low-prevalence IUs.

In the school-based survey conducted in 2021, a total of 1924 children were registered across the 11 low-prevalence schools. Of these 1924 children, 332 (17.3%) were absent on the day of urine collection, and 32 (1.7%) refused to participate or did not provide a signed consent form, resulting in 1560 (81.1%) children providing a fresh urine sample for examination (Supplementary File 1: Fig. 1A).

In the household-based survey in 2021, 3539 household members were registered across the 15 low-prevalence communities. Of these 3539 household members, 564 (15.9%) were absent on the day of urine collection, refused to participate, or did not provide a signed consent form. Hence, a total of 2975 (84.1%) household members provided a fresh urine sample for examination (Supplementary File 1: Fig. 1A).

In the school-based survey in 2022, a total of 1898 children were registered across the 11 low-prevalence schools. Of these 1898 children, 238 (12.5%) were absent on the day of urine collection, and 15 (0.8%) refused to participate or did not provide a signed consent form. Hence, 1645 (86.7%) children provided a fresh urine sample for examination (Supplementary File 1: Fig. 1B).

In the household-based survey in 2022, 3683 household members were registered across the 15 low-prevalence communities. Of these 3683 household members, one (0.03%) person was excluded due to ineligibility (age < 4 years), 755 (20.5%) household members were absent on the day of urine collection, and 7 (0.2%) individuals refused to participate or did not provide a signed consent form, resulting in 2920 (79.3%) household members providing a fresh urine sample (Supplementary File 1: Fig. 1B).

Demographic information from participants of all surveys and interventions is shown in Table 1.

**Table 1:** Demographic information of study participants. Demographic information of participants in school-based and household-based surveys and test-treat-track-test-treat (5T) activities implemented in 15 low *Schistosoma haematobium* prevalence implementation units in the North of Pemba, Tanzania, in 2021–2022

		2021 Parasitological survey				2021 Test-Treat-Track-Test-Treat intervention period										2022 Parasitological survey			
		2021 school-based survey		2021 household-based survey		2021 primary school testing		2021 madrassa testing		2021 household tracking		2021 water body tracking		2021 health facilities test-and-treat		2022 school-based survey		2022 household-based survey	
N		1560		2975		3704		594		258		60		354		1645		2920	
	*Female n (%)*	839	(53.8)	1570	(52.8)	1942	(52.4)	284	(47.8)	152	(58.9)	27	(45.0)	201	(56.8)	855	(52.0)	1617	(55.4)
	*Male n (%)*	721	(46.2)	1405	(47.2)	1762	(47.6)	310	(52.2)	106	(41.1)	33	(55.0)	153	(43.2)	790	(48.0)	1303	(44.6)
Age (median)		10		17		11		9		16.5		13		20		10		18	

### Participation in the 5T intervention period in 2021

**Fig. 1:**
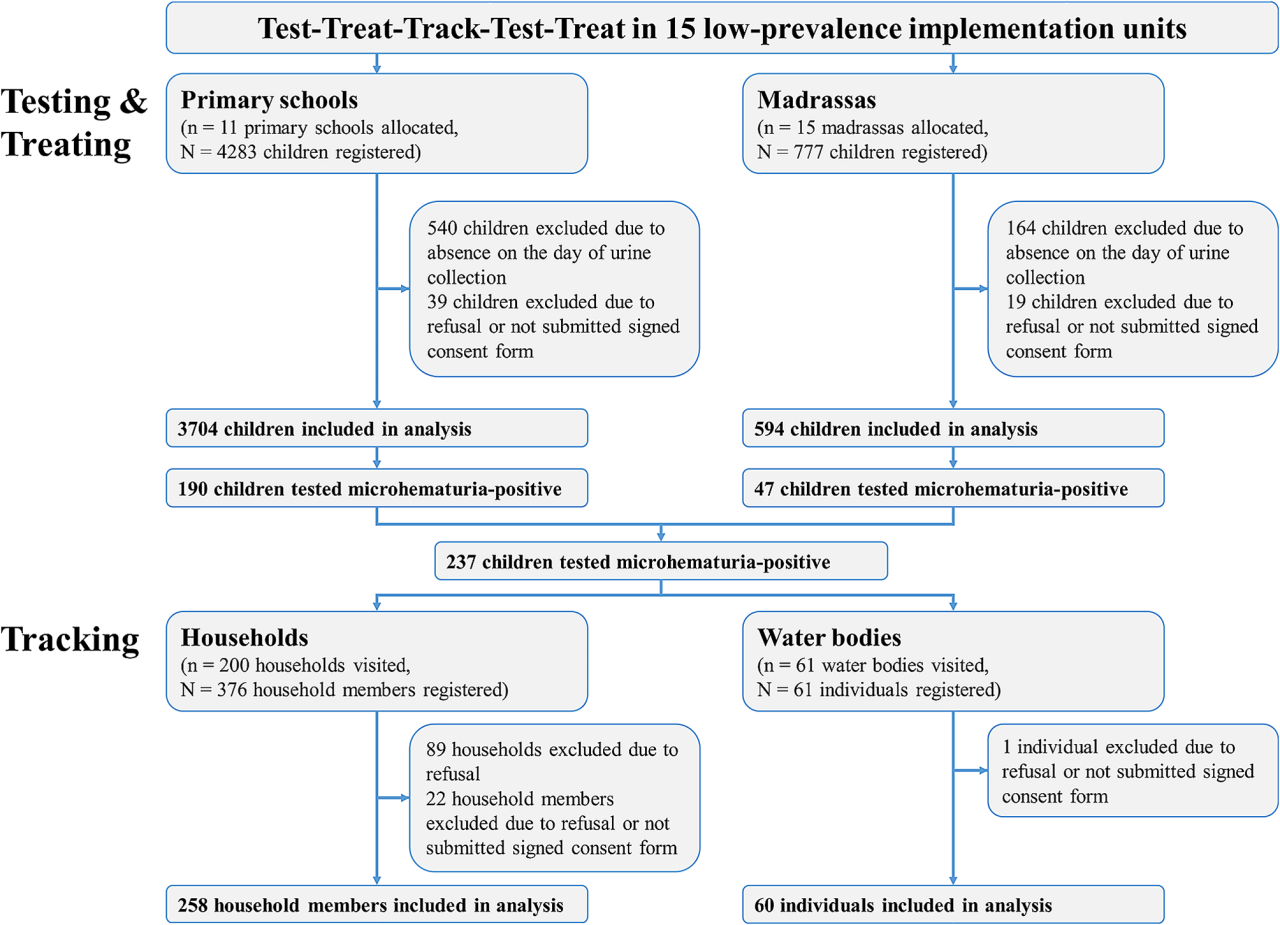
Participation in test-treat-track-test-treat interventions. Flow diagram of individuals participating in test-treat-track-test-treat interventions in 15 low *Schistosoma haematobium* prevalence implementation units in the North of Pemba, Tanzania, in 2021

Between May and October 2021, a total of 4283 and 777 children were registered in 11 schools and 15 madrassas, respectively, across the 15 low-prevalence IUs (Fig. 1). Of the 4283 children registered in the primary schools, 540 (12.6%) were absent on the day of urine collection and 39 (0.9%) refused to participate or did not provide a signed consent form. Hence, 3704 (86.5%) children were tested for microhematuria and included in the analysis. Of the 777 children registered in the madrassas, 164 (21.1%) were absent on the day of urine collection, and 19 (2.4%) refused to participate or did not provide a signed consent form. Hence, a total of 594 (76.4%) children were tested for microhematuria and included in the analysis.

Of the 3704 children tested in primary schools and 594 children tested in madrassas, 190 (5.1%) and 47 (7.9%), respectively, were microhematuria-positive.

Of the 237 microhematuria-positive children, 215 (90.7%) were tracked to 200 households. In 89 (44.5%) of the 200 households, nobody was present at the time of visit or all present members refused to participate. In the remaining 111 households, of the 280 household members present at the time of the visit, 22 (7.9%) refused to participate or did not provide a signed consent form. A total of 258 (92.1%) household members provided a fresh urine sample and had demographic data recorded.

Of the 237 microhematuria-positive children, 189 (79.7%) were tracked to 61 water bodies. At 15 water bodies, a total of 61 individuals were present at the time of the visit. Of the 61 individuals, one (1.6%) person refused to participate or did not provide a signed consent form. A total of 60 (98.4%) individuals provided a fresh urine sample and had demographic data recorded.

Information about sex and median age of the study participants, stratified per survey and intervention period, is indicated in Table 1.

### Microhematuria and ***Schistosoma haematobium*** prevalence in surveys, test-treat-track-test-treat intervention period, and health facilities

**Fig. 2:**
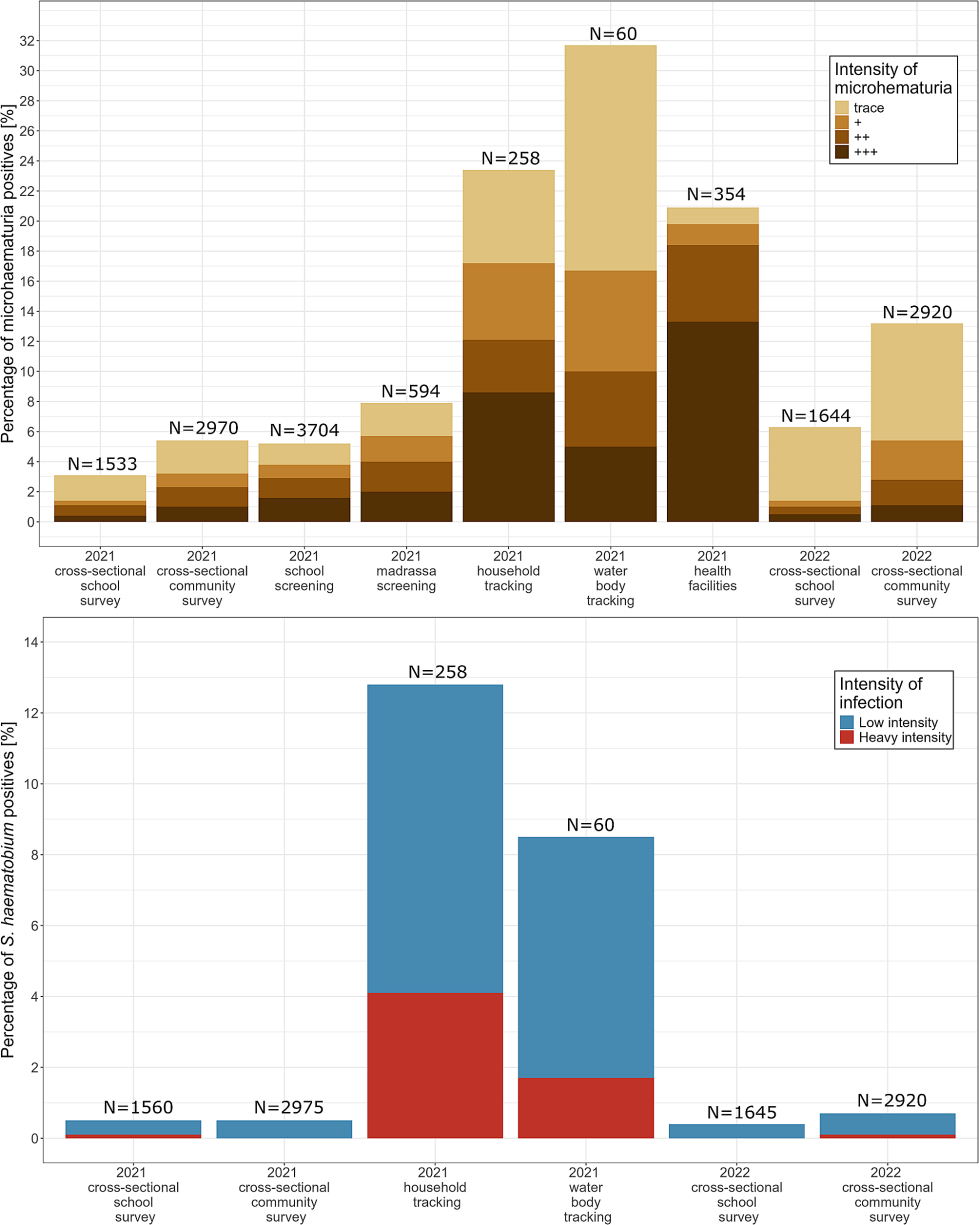
Microhematuria (**A**) and *Schistosoma haematobium* (**B**) prevalence in 15 low-prevalence implementation units in Pemba, Tanzania, in 2021–2022. The y-axis represents the percentage of positive-tested individuals for microhematuria (**A**) or *S. haematobium* infection (**B**). Colors indicate the grading of microhematuria or intensity of *S. haematobium* infections. N indicates the overall number of tested individuals per bar

The overall prevalence of microhematuria among children who participated in the school-based surveys in the 15 low-prevalence IUs was 3.1% (47/1533; 95% CI: 2.3–4.1) in 2021 and 6.3% (104/1644; 95% CI: 5.2–7.6) in 2022 (Fig. 2A and Supplementary File 2: Table 1). The results of the two-sample proportion test showed that the prevalence between the two years was significantly different (*p* < 0.0001). The prevalence of microhematuria among participants of the household-based survey was 5.5% (162/2970; 95% CI: 4.7–6.4) in 2021 and 13.2% (386/2920; 95% CI: 12.0–14.5) in 2022. The results of the two-sample proportion test showed that the prevalence between the two years was significantly different (*p* < 0.0001).

The prevalence of microhematuria excluding trace results among children who participated in the school-based surveys in the 15 low-prevalence IUs was 1.4% (21/1533; 95% CI: 0.9–2.1) in 2021 and 1.5% (24/1644; 95% CI: 1.0–2.2) in 2022. The results of the two-sample proportion test showed that the prevalence between the two years was not significantly different (*p* = 0.93). The prevalence of microhematuria among participants of the household-based survey was 3.3% (98/2975; 95% CI: 2.7–4.0) in 2021 and 5.4% (159/2920; 95% CI: 4.6–6.3) in 2022. The results of the two-sample proportion test showed that the prevalence between the two years was significantly different (*p* < 0.0001).

Figure 2B shows that the overall *S. haematobium* prevalence, as assessed in the school-based surveys, was 0.5% (7/1560; 95% CI: 0.2–1.0) in 2021 and 0.4% (6/1645; 95% CI: 0.2–0.9) in 2022 in schoolchildren attending the 11 primary schools of the 15 low-prevalence IUs. The results of the two-sample proportion test showed that the prevalence between the two years was not significantly different (*p* = 0.87). The *S. haematobium* prevalence, assessed in the household-based survey in the communities of the 15 low-prevalence IUs, was 0.5% (14/2975; 95% CI: 0.3–0.8) in 2021 and 0.7% (19/2920; 95% CI: 0.4–1.1) in 2022. The results of the two-sample proportion test showed that the prevalence between the two years was not significantly different (*p* = 0.41).

During the 5T intervention period in 2021, 5.1% (190/3704) of the children tested in primary schools and 8.0% (47/594) of the children tested in madrassas were microhematuria-positive (Fig. 2A). Of the 190 and 47 children tested positive for microhematuria in schools and madrassas, 180 (95.0%) and 42 (89.4%), respectively, were treated with praziquantel after testing. In the households and at the water bodies identified by tracking these microhematuria-positive children, 23.3% (60/258) and 31.7% (19/60), respectively, of the tested individuals were microhematuria-positive. Of the 60 and 19 individuals who tested positive in households and at water bodies, 98.3% (59/60) and 94.7% (18/19), respectively, were treated at the point-of-care.

The risk-based tracking of microhematuria-positive children from primary schools or madrassas to their homes identified 12.8% (31/243) of household members present at home at the time of the visit as *S. haematobium* egg-positive (Fig. 2B). At the water bodies that were identified by tracking microhematuria-positive children, 8.5% (5/60) of individuals present at the water bodies at the time of the visit were tested *S. haematobium* egg-positive.

Between August and November 2021, 354 patients presented with symptoms aligning with urogenital schistosomiasis in 13 of the 22 health facilities in the study area. All 354 individuals were tested with Hemastix reagent strips at the point-of-care. Among them, 20.9% (74/354) were microhematuria-positive (Fig. 2A). Of these 74 individuals, 90.5% (67/74) were treated with praziquantel in the health facilities.

Information on microhematuria and *S. haematobium* prevalence for each of the surveyed, tested and tracked populations, stratified by sex, are presented in Supplementary File 2: Table 1.

### Heterogeneity of microhematuria and ***S. haematobium*** prevalence during the test-treat-track-test-treat intervention period

**Fig. 3:**
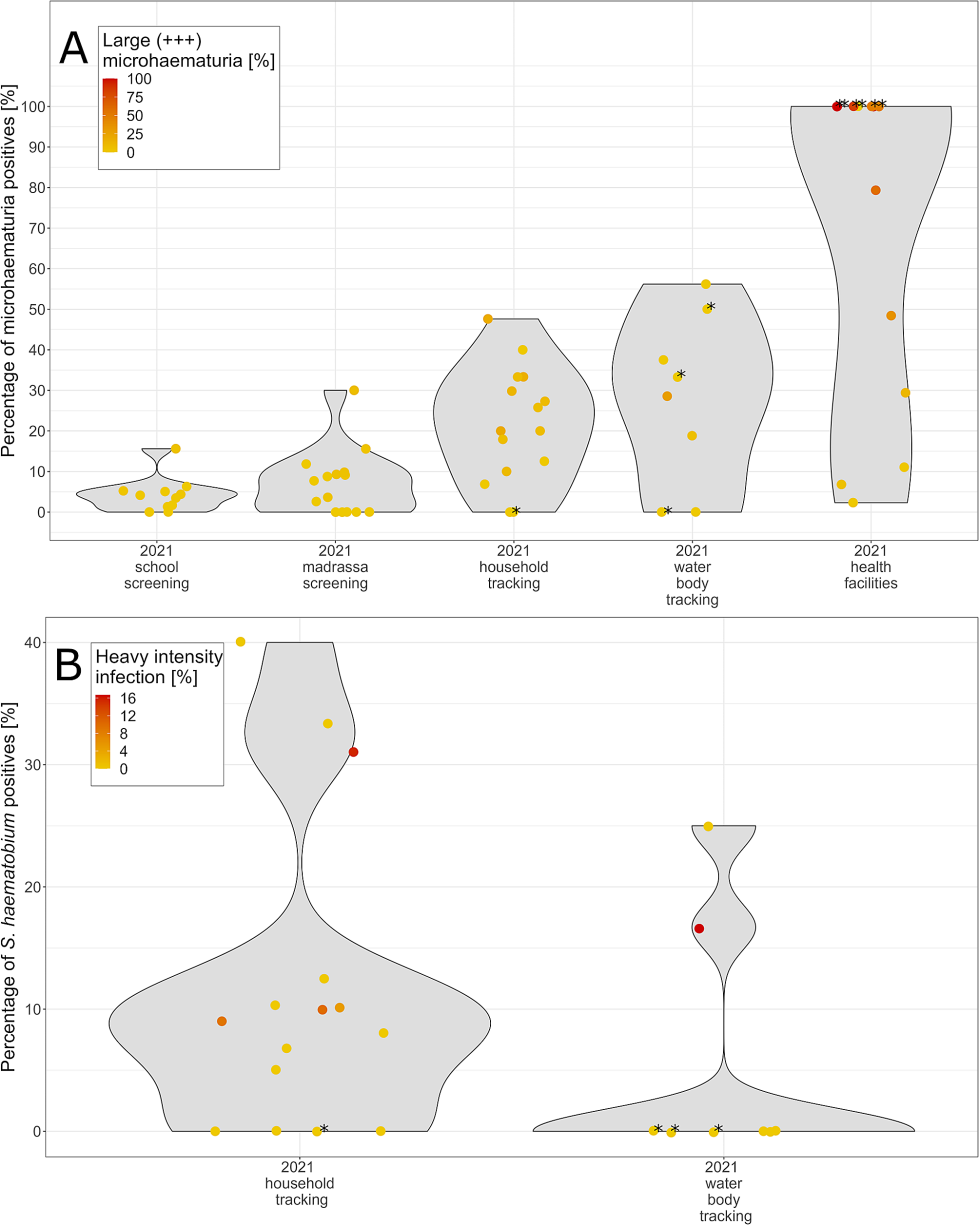
Microhematuria (**A**) and *Schistosoma haematobium* (**B**) prevalence in 15 low-prevalence implementation units in Pemba, Tanzania, in 2021. The y-axis represents the prevalence of microhematuria (**A**) or *S. haematobium* infection (**B**); each point represents one school/community. Colors indicate the grading of microhematuria or *S. haematobium* intensity infections, respectively, per school/community. The asterisk (*) denotes points where the prevalence was calculated with < 5 participants per school/community

The testing during the 5T intervention period showed a varying microhematuria prevalence between 0.0% and 15.6% in schools and between 0.0% and 30.0% in madrassas (Fig. 3A). The school and madrassa with the highest microhematuria prevalence were located in the same IU. During the risk-based tracking of children who tested positive for microhematuria in schools and madrassas, the microhematuria prevalence per IU varied between 0.0% and 47.6% in households and between 0.0% and 56.2% at water bodies, respectively.

In the 5T intervention period, the *S. haematobium* prevalence per IU ranged from 0.0 to 40.0% in households and from 0.0 to 25.0% at the water bodies, respectively (Fig. 3B).

In the test-and-treat intervention in the health facilities, the microhematuria prevalence ranged from 0.0 to 100% in health facilities (Fig. 3A). However, it should be noted that all health facilities with a microhematuria prevalence of 100% had tested < 5 patients with urogenital schistosomiasis-related symptoms.

### Children tested in madrassas during the test-treat-track-test-treat activities

Of the 594 madrassa students who were tested for microhematuria, 219 (36.9%) attended a primary school that was part of our 5T interventions, but were enrolled in grades where screening for microhematuria was not operated, i.e., in nursery school, grades 1, 2, or grade 6. Another 79 (31.3%) of the 594 tested madrassa students reported that they would not visit a school. For 132 (22.2%) children, no information was available on their primary/secondary school enrolment and attendance because the assessment of primary school enrolment was only started from the third madrassa onwards. Another 97 (16.3%) of the 594 madrassa students were enrolled in primary schools that were not part of the SchistoBreak study schools. A total of 51 (8.6%) children were enrolled in grades 3–5 in one of the SchistoBreak study schools but were not present on the testing days, and 16 (2.7%) children were enrolled in secondary schools, which were not part of our 5T interventions. Of the 79 non-school attendees, 5 (6.3%) children were tested microhaematuria-positive. The microhaematuria prevalence in school attendees tested in madrassas was 9.1% (35/383).

### Households and water bodies identified during the test-treat-track-test-treat interventions

**Fig. 4:**
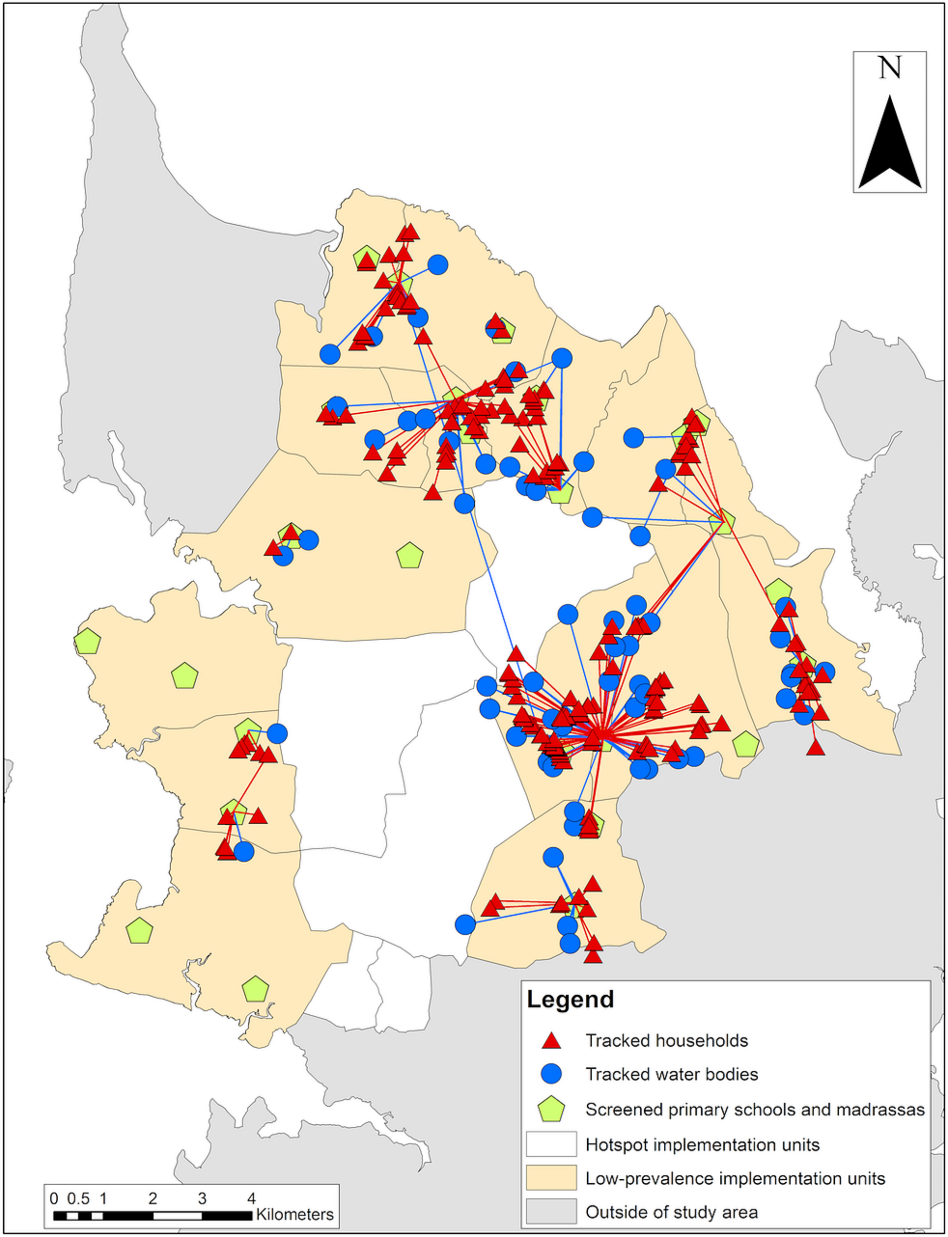
Spatial distribution of tracked households and water bodies. Spatial distribution of households of microhematuria-positive children and water bodies used and indicated by microhematuria-positive children emanating from the school/madrassa where the children were tested in the 15 low S*chistosoma haematobium* prevalence implementation units in the North of Pemba, Tanzania, in 2021. The locations of households are geographically masked in order to preserve confidentiality

The households of microhematuria-positive children were located between 8 and 4794 m (mean: 958 m) from the school or madrassa where the children were tested. Of the 215 children who were tracked to their homes, 71 (32.0%) children lived in households not located in the same IU as the school the children were attending (Fig. 4).

The water bodies indicated by microhematuria-positive children were located between 62 m and 10.1 km (mean: 1210 m) away from the school or madrassa where the children were tested. For 27.2% (43/158) of the positive-tested children, the water bodies they frequented were not located in the same IU as the screened school. Moreover, several water bodies were located at borders between two IUs and borders to shehias outside of the study area.

In two schools and five madrassas, respectively, no child tested positive for microhematuria, and therefore, no child was tracked to household or water bodies.

### Individuals treated and praziquantel tablets used during the mass drugs administration in 2020 and the 5T interventions in 2021

During the MDA in August 2020, 39,312 individuals were treated with praziquantel in the communities and schools of the 15 IUs that were identified as low-prevalence IUs in 2021 (Table 2). To treat these individuals, 78,853 praziquantel tablets were used, with an average of 2 tablets per treated person. During the 5T interventions in 2021, 336 individuals were treated in primary schools, madrassas, at households and at water bodies in the 15 low-prevalence IUs. For the 336 individuals, 1123 praziquantel tablets were used, with an average of 3 tablets per treated person. Hence, during the MDA in August 2020 around 70 times as many praziquantel tablets were used as during the targeted 5T interventions in 2021 in the same IUs. An additional 85 praziquantel tablets were used to treat 31 hematuria-positive individuals in the health facilities across the study area.

**Table 2:** Praziquantel tablets administered during mass drug administration (MDA) in August 2020 and during targeted test-and-treat interventions in 2021. Number of praziquantel tablets administered during the MDA in August 2020 and the test-treat-track-test-treat (5T) intervention period from May to October 2021 and number of praziquantel tablets used during test-and-treat activities in health facilities from July to November 2021 in 15 low *Schistosoma haematobium* prevalence implementation units in the north of Pemba, Tanzania. NA = not applicable because the information is missing. “-“ indicates target points where MDA was not planned and conducted

District(s)	Location of treatment interventions	Number of praziquantel tablets used in MDA in August 2020	Number of individuals treated in MDA in August 2020	Number of praziquantel tablets used in 5T and test-and-treat interventions in 2021	Number of individuals treated in 5T and test-and-treat interventions in 2021
Wete	Primary schools	6316	3378	239.5	110
	Madrassas	NA	NA	103	53
	Households	11,290	3924	22.5	8
	Water bodies	-	-	10	12
Micheweni	Primary schools	18,718	10,765	544	53
	Madrassas	NA	NA	70.5	43
	Households	42,529	21,245	120.5	51
	Water bodies	-	-	13	6
	**Total**	**78,853**	**39,312**	**1123**	**336**
Wete and Micheweni	Health facilities	-	-	85	31
	**Total**	**78,853**	**39,312**	**1208**	**367**

## Discussion

The Zanzibar islands have achieved the elimination of urogenital schistosomiasis as a public health problem in 2017 and have aimed for interruption of transmission ever since [11, 13]. Considering that in many communities on the islands the majority of people are not infected with *S. haematobium*, large-scale praziquantel MDA seems no longer justified. Alternative intervention strategies that maintain the gains made by MDA over the past decades and ideally allow Zanzibar to accelerate elimination need to be explored.

Over the first year of the SchistoBreak study, we assessed whether targeted 5T interventions were able to maintain or even reduce the prevalence of urogenital schistosomiasis in 15 low-prevalence shehias in the north of Pemba. Our results showed no considerable change in the prevalence of microhematuria (excluding trace results) and of *S. haematobium* infections after one year of 5T interventions from 2021 to 2022. Hence, aligned with targeted snail control measures that were also part of the surveillance-response activities [17], the 5T interventions were able to maintain the very low overall prevalence, and no outbreaks or major recrudescence were observed. However, the prevalence of microhematuria including trace increased significantly from 2021 to 2022 in the school-based and community-based surveys. While this observation warrants close monitoring in the following study years, since increasing (trace) microhematuria might be a first sign of infections and recurrent morbidity in individuals missed by the 5T interventions, our current assumption is that the increase in trace microhematuria was caused by different and newer batches of Hemastix that were started to be used after the baseline survey in 2021. Other studies from Pemba and Tanzania mainland confirm that older/expired strips have a less intense color reaction [24] and recommend that trace results should be considered negative for *S. haematobium* infections when a high specificity is aimed for in settings with low egg counts in urine filtration [25].

With certainty, however, we can say that the 5T interventions did not contribute to further reducing the overall microhematuria or *S. haematobium* prevalence in our study within one year. Whether the 5T approach is an appropriate intervention to accelerate elimination and progress towards interruption of transmission remains in question, and more evidence needs to be generated in long-term studies. Close monitoring and regular evaluation is crucial once MDA is stopped and alternative targeted interventions are applied, to allow a timely reaction in case of any sign of recrudescence [26].

In our study, the 5T interventions were an excellent and straightforward approach to identifying many of the few *S. haematobium*-infected individuals in schools, in households of microhematuria-positive children, and at water bodies used by these children. Compared with the very low overall prevalence in schools and communities, a percentage of 12.8% of household members that were infected with *S. haematobium* confirmed the assumption that household members of positive children are also likely to be infected since they may use the same water bodies where transmission occurs. Moreover, the freshwater bodies that microhematuria-positive children reported to use were ideal places to identify and treat additional infected individuals: a percentage of 8.5% of people using these water bodies tested positive for *S. haematobium*. Our results are in line with a study that assessed similar 5T interventions for *S. mansoni* control in two villages in mainland Tanzania, which revealed an even higher percentage (46.8%) of *S. mansoni* infections among household members of positive-tested children [27]. Since this study used a prevalence threshold of < 10% to define a low-prevalence IU, the research team was likely working in a setting with a considerably higher prevalence than that in Pemba, and the higher percentage of infected household members is not surprising. The study team from mainland Tanzania also tracked friends of *S. mansoni*-positive children and their household members and identified 37.5% of the friends and 47.1% of the friends’ household members as infected, showing that testing friends and their close contacts can be another important supplement when recommending 5T interventions for implementation.

In addition to public primary schools, madrassas proved to be useful venues for the initial testing of children. While more than one third of the children who attended our study madrassas were also enrolled in our study primary schools, they attended grades that were not included in our testing. These students could have also been reached through extended testing in primary schools, which we did not have the capacity and resources to do. However, another third of the tested madrassa students did not attend a primary school, either because they were too young or for other unknown reasons. Thus, as suggested for behavior change communication about schistosomiasis [28], madrassas offer a fantastic opportunity to include non-school attendees into 5T interventions who otherwise would not have been reached but who may have a strong exposure to the parasite during their daily activities. The microhematuria prevalence in non-school attendees and school attendees tested in madrassas was 6.3% and 9.1%, respectively. Hence, while non-school attendees seemed not at higher risk for obtaining a *S. haematobium* infection than school attendees in our study, they are yet an important group for inclusion in 5T interventions.

In our study, with a team of four enumerators, more than 4000 children were tested in five months, and 200 microhematuria-positive children were tracked to their households and the water bodies they had used. Since the mean distance between the schools/madrassas and households or frequented water bodies was only about 1000 m, tracking children to these points was mostly easily conducted. A challenge during the 5T interventions, however, was the considerably low number of individuals who were present at the water bodies at times when the study teams visited, in addition to only few individuals who agreed to participate in test-and-treat activities at households and at water bodies. To reach more people with 5T interventions, good outreach, and awareness campaigns to increase health literacy about urogenital schistosomiasis are needed in target areas. Moreover, it may be worth returning to the same water body several times and staying there for an extended period, as was done in a study in Egypt where the enumerators spent 11 consecutive hours at tracked water bodies to offer test-and-treat to present or arriving individuals [12]. Working with a larger team or multiple teams in our study would undoubtedly have allowed us to test an even larger number of children and schools, to visit households and water bodies at more ideal times and for longer hours, to conduct multiple rounds of testing per school, and to follow-up treated individuals in a similar time frame. This would have increased the overall coverage of test-and-treat and improved treatment outcomes and would hence potentially have resulted in better progress towards interruption of transmission. However, more staff and diagnostic material have cost implications and our financial resources were limited.

Test-and-treat interventions were also offered by health facilities in the study area to patients with symptoms consistent with a *S. haematobium* infection. In health facilities, patients were tested exclusively with Hemastix reagent strips, and the very high percentage of microhematuria in the patients (20.9%) compared with microhematuria levels we found in community surveys indicates that health facilities may offer another important location for reaching and treating infected individuals. For this purpose, however, it needs to be ensured that health facilities have testing equipment and can provide access to praziquantel as recommended by WHO [3, 29]. Microhematuria-positive patients from our study health facilities were not tracked, in contrast to another study from Egypt, where health facility patients with *S. mansoni* infection were tracked, and their household members were tested for schistosomiasis and treated if positive [12]. Also in malaria surveillance research and practice, tracking malaria patients identified in health facilities to their homes and testing and treating household members and surrounding households is a renowned approach [30, 31]. In future schistosomiasis elimination activities, if resources allow, it may be worthwhile to incorporate the tracking, testing, and treating of household members of individuals who tested positive at health facilities to increase the coverage further.

As with MDA and any other intervention, good coverage is key to success. But resources are limited, and careful decisions have to be made about where to save and where to spend to ensure a cost-effective approach. What our study clearly showed, in addition to the other results, was that the number of praziquantel tablets required during the 5T interventions to treat infected individuals in schools, households, water bodies, and health facilities was much lower than the number of praziquantel tablets required during an MDA. These findings suggest that, by avoiding recrudescence and preventing an overtreatment of a mostly healthy population, 5T interventions may be considered a cost-effective and feasible alternative to MDA to maintain current gains in very low-prevalence settings. However, solid evidence on cost-effectiveness yet remains to be generated.

The inexistence of a sensitive and specific point-of-care test for *S. haematobium* diagnosis was a considerable challenge for our 5T approach. We used a combination of reagent strip and urine filtration tests conducted on a single urine sample to allow a maximum sensitivity to ensure that infected individuals were detected and treated. However, since the sensitivity of both tests to detect light intensity infections is low [32–34], several infected individuals may have been missed. Conversely, since the specificity of reagent strips to diagnose urogenital schistosomiasis is also low, especially in low-prevalence settings [33, 35], uninfected individuals may have been treated and tracked. Hence, the timely development of highly sensitive and specific point-of-care tests for the diagnosis of schistosomiasis (and other NTDs) in elimination settings is crucial to focus all efforts on true positives and thus further enhance the cost-effectiveness of the 5T interventions [36, 37].

## Conclusions

The 5T interventions described here were able to maintain the very low *S. haematobium* prevalence in our study IUs in the north of Pemba, and no outbreaks or major recrudescence were observed over the first year of implementation. While it remains in question whether it will expedite the interruption of transmission, the 5T interventions proved to be a straightforward and feasible approach to identifying and treating many of the few *S. haematobium*-infected individuals in an elimination setting. In addition to schools, madrassas were an excellent starting point for testing a high-risk population, including non-school attendees, which would then lead us to additional individuals with a high likelihood of exposure and infection in households and at water bodies. Health facilities were also an important venue for testing and treating patients with symptoms of urogenital schistosomiasis and confirmed microhematuria. Essential for a successful and cost-effective implementation of 5T interventions in elimination settings in the future will be the development of a highly sensitive and specific rapid diagnostic test that can be used at the point-of-care and enables a timely and accurate treatment and tracking of infected individuals. Close monitoring of the endemic situation after MDA has been stopped will be key to allow a timely reaction to recrudescence. More long-term and large-scale studies assessing the feasibility, impact and cost-effectiveness of 5T interventions in different prevalence and environmental settings are needed before it can be recommended to schistosomiasis elimination programs as an alternative or successor to MDA. Most importantly, it remains to be clarified whether 5T interventions are indeed capable of maintaining the gains made by control programs over extended periods and whether or not they can support programs that have the ultimate aim to interrupt *Schistosoma* transmission.

### Electronic supplementary material

Below is the link to the electronic supplementary material.

**Supplementary Material 1: Figure 1.** Flow diagram of individuals participating in the school-based and household-based surveys in low *Schistosoma haematobium* prevalence shehias on Pemba, Tanzania, in 2021 (A) and 2022 (B)

**Supplementary Material 2: Table 1.** Prevalence and intensity of microhematuria and *Schistosoma haematobium* infections of participants in school-based and household-based surveys 2021 and 2022, and test-treat-track-test-treat (5T) activities in 2021. NA = Not applicable

## Data Availability

Data are provided within the manuscript or supplementary information files.

## Abbreviations

5T: Test-treat-track-test-treat
IU: Implementation unit
MDA: Mass drug administration
NTD: Neglected tropical disease
ODK: Open Data Kit
PHCU: Primary health care unit
PHL-IdC: Public Health Laboratory – Ivo de Carneri
Swiss TPH: Swiss Tropical and Public Health Institute
WHO: World Health Organization
