# Supplementary material for: Test-treat-track-test-treat (5T) approach for Schistosoma haematobium elimination on Pemba Island, Tanzania
Source: BMC Infect Dis. 2024 Jul 2;24:661. doi: 10.1186/s12879-024-09549-w (PMC11218394; doi:10.1186/s12879-024-09549-w)
Supplement: Supplementary file 1 — Supplementary Material 1: Figure 1. Flow diagram of individuals participating in the school-based and household-based surveys in low Schistosoma haematobium prevalence shehias on Pemba, Tanzania, in 2021 (A) and 2022 (B) [file 12879_2024_9549_MOESM1_ESM.pdf]

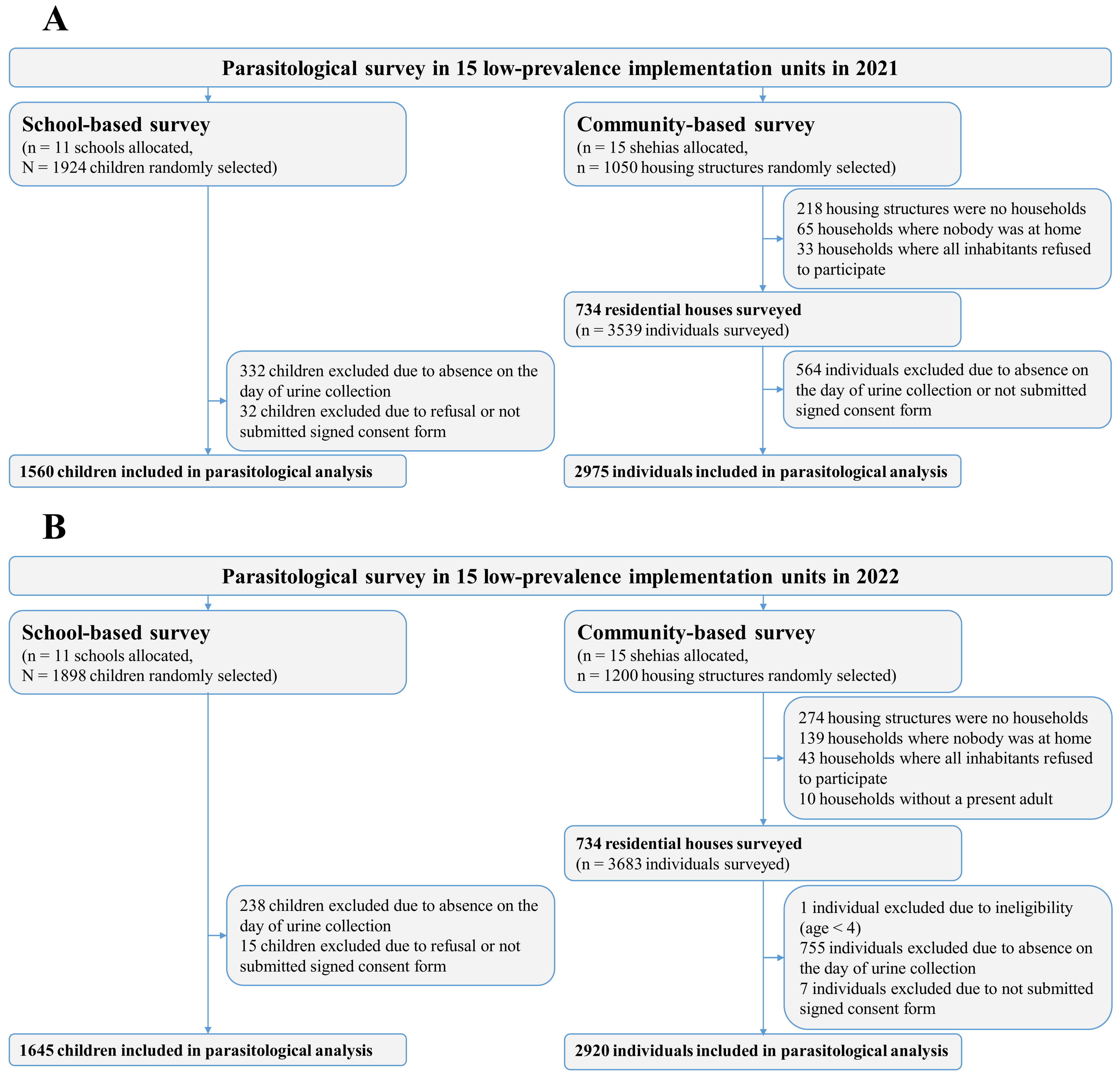
A
Parasitological survey in 15 low-prevalence implementation units in 2021
School-based survey
(n = 11 schools allocated,
N = 1924 children randomly selected)
Community-based survey
(n = 15 shehias allocated,
n = 1050 housing structures randomly selected)
218 housing structures were no households
65 households where nobody was at home
33 households where all inhabitants refused to participate
734 residential houses surveyed
(n = 3539 individuals surveyed)
332 children excluded due to absence on the day of urine collection
32 children excluded due to refusal or not submitted signed consent form
564 individuals excluded due to absence on the day of urine collection or not submitted signed consent form
1560 children included in parasitological analysis
2975 individuals included in parasitological analysis
B
Parasitological survey in 15 low-prevalence implementation units in 2022
School-based survey
(n = 11 schools allocated,
N = 1898 children randomly selected)
Community-based survey
(n = 15 shehias allocated,
n = 1200 housing structures randomly selected)
274 housing structures were no households
139 households where nobody was at home
43 households where all inhabitants refused to participate
10 households without a present adult
734 residential houses surveyed
(n = 3683 individuals surveyed)
238 children excluded due to absence on the day of urine collection
15 children excluded due to refusal or not submitted signed consent form
1 individual excluded due to ineligibility (age < 4)
755 individuals excluded due to absence on the day of urine collection
7 individuals excluded due to not submitted signed consent form
1645 children included in parasitological analysis
2920 individuals included in parasitological analysis
