# Supplementary material for: Test-treat-track-test-treat (5T) approach for Schistosoma haematobium elimination on Pemba Island, Tanzania
Source: BMC Infect Dis. 2024 Jul 2;24:661. doi: 10.1186/s12879-024-09549-w (PMC11218394; doi:10.1186/s12879-024-09549-w)
Supplement: Supplementary file 2 — Supplementary Material 2: Table 1. Prevalence and intensity of microhematuria and Schistosoma haematobium infections of participants in school-based and household-based surveys 2021 and 2022, and test-treat-track-test-treat (5T) activities in 2021. NA = Not applicable [file 12879_2024_9549_MOESM2_ESM.pdf]

		**2021 school-based survey**		**2021 household-based survey**		**2021 school testing**		**2021 madrassa testing**		**2021 household tracking**		**2021 water body tracking**		**2021 health facilities**		**2022 school-based survey**		**2022 household-based survey**	
N		1560		2975		3700		594		258		60		354		1645		2920	
	female	839		1570		1942		284		152		27		201		855		1617	
	male	721		1405		1758		310		106		33		153		790		1303	
Age (median)		10		17		11		9		16.5		13		20		10		18	
Microhaematuria positive, n (%)		47	(3.1)	162	(5.5)	190	(5.1)	47	(8.0)	60	(23.3)	19	(31.7)	74	(20.9)	104	(6.3)	386	(13.2)
	female	27		110		105		27		32		11		36		55		249	
	male	20		52		85		20		28		8		38		49		137	
Age (median)		10		23		12		8		11		20		22		11		21	
Trace of microhaematuria, n (%)		26	(1.7)	64	(2.2)	50	(1.4)	13	(2.2)	16	(6.2)	9	(15.0)	4	(1.1)	80	(4.9)	227	(7.8)
Small microhaematuria (+), n (%)		5	(0.3)	28	(0.9)	33	(0.9)	10	(1.7)	13	(5.1)	4	(6.7)	5	(1.4)	7	(0.4)	77	(2.6)
Moderate microhaematuria (++), n (%)		10	(0.7)	39	(1.3)	47	(1.3)	12	(2.0)	9	(3.5)	3	(5.0)	18	(5.1)	8	(0.5)	49	(1.7)
Large microhaematuria (+++), n (%)		6	(0.4)	31	(1.0)	60	(1.6)	12	(2.0)	22	(8.6)	3	(5.0)	47	(13.3)	9	(0.5)	33	(1.1)
Urine filtration positive, n (%)		7	(0.5)	14	(0.5)	NA		NA		31	(12.8)	5	(8.5)	NA		6	(0.4)	19	(0.7)
	female	3		8		NA		NA		11		1		NA		2		9	
	male	4		6		NA		NA		20		4		NA		4		10	
Age (median)		9		10.5		NA		NA		8		18		NA		11		16	
Urine filtration heavy intensity, n (%)		2	(0.1)	1	(0.0)	NA		NA		10	(4.1)	1	(1.7)	NA		0	(0.0)	2	(0.1)
